# Supplementary material for: Modeling glioblastoma heterogeneity as a dynamic network of cell states
Source: Mol Syst Biol. 2021 Sep 16;17(9):e10105. doi: 10.15252/msb.202010105 (PMC8444284; doi:10.15252/msb.202010105)
Supplement: Supplementary file 6 — Source Data for Figure 5 [file MSB-17-e10105-s004.zip › Figure5A_sourcedata/GSEA_3071/hallmarks_stateV.GseaPreranked.1623742546329/HALLMARK_P53_PATHWAY.html]

Details for gene set HALLMARK\_P53\_PATHWAY[GSEA]

|  || Dataset | state43071 |
| Phenotype | NoPhenotypeAvailable |
| Upregulated in class | na\_neg |
| GeneSet | HALLMARK\_P53\_PATHWAY |
| Enrichment Score (ES) | -0.37919685 |
| Normalized Enrichment Score (NES) | -2.1080215 |
| Nominal p-value | 0.0 |
| FDR q-value | 4.7160996E-4 |
| FWER p-Value | 0.004 |
Table: GSEA Results Summary

  

Fig 1: Enrichment plot: HALLMARK\_P53\_PATHWAY      
 Profile of the Running ES Score & Positions of GeneSet Members on the Rank Ordered List

  

| PROBE | GENE SYMBOL | GENE\_TITLE | RANK IN GENE LIST | RANK METRIC SCORE | RUNNING ES | CORE ENRICHMENT || 1 | CCP110 |  |  | 9 | 1.920 | 0.0257 | No |
| 2 | PCNA |  |  | 10 | 1.908 | 0.0530 | No |
| 3 | CDH13 |  |  | 185 | 1.204 | 0.0368 | No |
| 4 | CD82 |  |  | 208 | 1.163 | 0.0492 | No |
| 5 | RGS16 |  |  | 348 | 1.018 | 0.0370 | No |
| 6 | CDKN1A |  |  | 363 | 0.993 | 0.0485 | No |
| 7 | APP |  |  | 570 | 0.818 | 0.0206 | No |
| 8 | CYFIP2 |  |  | 621 | 0.788 | 0.0223 | No |
| 9 | NOTCH1 |  |  | 673 | 0.762 | 0.0233 | No |
| 10 | NINJ1 |  |  | 695 | 0.748 | 0.0300 | No |
| 11 | HSPA4L |  |  | 793 | 0.703 | 0.0214 | No |
| 12 | POLH |  |  | 807 | 0.697 | 0.0289 | No |
| 13 | RB1 |  |  | 810 | 0.696 | 0.0384 | No |
| 14 | DDB2 |  |  | 872 | 0.669 | 0.0363 | No |
| 15 | MDM2 |  |  | 904 | 0.658 | 0.0397 | No |
| 16 | FBXW7 |  |  | 1002 | 0.620 | 0.0299 | No |
| 17 | BAX |  |  | 1030 | 0.613 | 0.0335 | No |
| 18 | TRAF4 |  |  | 1069 | 0.602 | 0.0348 | No |
| 19 | RAD9A |  |  | 1083 | 0.597 | 0.0409 | No |
| 20 | PIDD1 |  |  | 1092 | 0.594 | 0.0478 | No |
| 21 | TM7SF3 |  |  | 1113 | 0.589 | 0.0524 | No |
| 22 | ZMAT3 |  |  | 1129 | 0.585 | 0.0579 | No |
| 23 | PPM1D |  |  | 1419 | 0.498 | 0.0094 | No |
| 24 | TGFA |  |  | 1616 | 0.457 | -0.0217 | No |
| 25 | FDXR |  |  | 1688 | 0.438 | -0.0291 | No |
| 26 | SP1 |  |  | 1874 | 0.407 | -0.0589 | No |
| 27 | WWP1 |  |  | 1916 | 0.401 | -0.0610 | No |
| 28 | RALGDS |  |  | 1950 | 0.396 | -0.0617 | No |
| 29 | CCND3 |  |  | 1991 | 0.390 | -0.0638 | No |
| 30 | ABAT |  |  | 2170 | 0.365 | -0.0928 | No |
| 31 | XPC |  |  | 2211 | 0.361 | -0.0954 | No |
| 32 | LDHB |  |  | 2219 | 0.360 | -0.0916 | No |
| 33 | AEN |  |  | 2315 | 0.347 | -0.1049 | No |
| 34 | TOB1 |  |  | 2442 | 0.330 | -0.1244 | No |
| 35 | PITPNC1 |  |  | 2468 | 0.326 | -0.1245 | No |
| 36 | SDC1 |  |  | 2491 | 0.323 | -0.1241 | No |
| 37 | APAF1 |  |  | 2504 | 0.322 | -0.1218 | No |
| 38 | FOS |  |  | 2529 | 0.319 | -0.1219 | No |
| 39 | IP6K2 |  |  | 2645 | 0.305 | -0.1397 | No |
| 40 | DCXR |  |  | 2647 | 0.305 | -0.1355 | No |
| 41 | WRAP73 |  |  | 2786 | 0.291 | -0.1579 | No |
| 42 | CCND2 |  |  | 2876 | 0.282 | -0.1710 | No |
| 43 | CDK5R1 |  |  | 2919 | 0.277 | -0.1751 | No |
| 44 | TSC22D1 |  |  | 2978 | 0.272 | -0.1823 | No |
| 45 | PTPRE |  |  | 3014 | 0.267 | -0.1852 | No |
| 46 | PLXNB2 |  |  | 3068 | 0.262 | -0.1917 | No |
| 47 | STOM |  |  | 3230 | -0.256 | -0.2190 | No |
| 48 | CCNK |  |  | 3257 | -0.260 | -0.2203 | No |
| 49 | RHBDF2 |  |  | 3281 | -0.263 | -0.2209 | No |
| 50 | TRIAP1 |  |  | 3300 | -0.266 | -0.2206 | No |
| 51 | RAP2B |  |  | 3360 | -0.276 | -0.2280 | No |
| 52 | HMOX1 |  |  | 3426 | -0.286 | -0.2364 | No |
| 53 | STEAP3 |  |  | 3644 | -0.321 | -0.2735 | No |
| 54 | CASP1 |  |  | 3648 | -0.323 | -0.2695 | No |
| 55 | VDR |  |  | 3793 | -0.348 | -0.2922 | No |
| 56 | BAIAP2 |  |  | 3844 | -0.357 | -0.2967 | No |
| 57 | KLF4 |  |  | 3866 | -0.361 | -0.2956 | No |
| 58 | RPS27L |  |  | 3927 | -0.373 | -0.3018 | No |
| 59 | RPL36 |  |  | 3952 | -0.377 | -0.3010 | No |
| 60 | BLCAP |  |  | 3972 | -0.383 | -0.2992 | No |
| 61 | OSGIN1 |  |  | 3996 | -0.388 | -0.2981 | No |
| 62 | RACK1 |  |  | 4022 | -0.393 | -0.2973 | No |
| 63 | JUN |  |  | 4135 | -0.418 | -0.3128 | No |
| 64 | KIF13B |  |  | 4153 | -0.425 | -0.3100 | No |
| 65 | DDIT4 |  |  | 4182 | -0.432 | -0.3092 | No |
| 66 | CDKN2A |  |  | 4301 | -0.472 | -0.3252 | No |
| 67 | F2R |  |  | 4316 | -0.476 | -0.3210 | No |
| 68 | GADD45A |  |  | 4399 | -0.500 | -0.3297 | No |
| 69 | RPL18 |  |  | 4532 | -0.555 | -0.3471 | No |
| 70 | TXNIP |  |  | 4618 | -0.604 | -0.3548 | No |
| 71 | TAX1BP3 |  |  | 4631 | -0.609 | -0.3484 | No |
| 72 | MKNK2 |  |  | 4792 | -0.706 | -0.3691 | Yes |
| 73 | HBEGF |  |  | 4822 | -0.734 | -0.3642 | Yes |
| 74 | SLC3A2 |  |  | 4861 | -0.765 | -0.3606 | Yes |
| 75 | LIF |  |  | 4882 | -0.789 | -0.3531 | Yes |
| 76 | RPS12 |  |  | 4888 | -0.795 | -0.3427 | Yes |
| 77 | MXD4 |  |  | 4915 | -0.825 | -0.3359 | Yes |
| 78 | ZFP36L1 |  |  | 4929 | -0.843 | -0.3263 | Yes |
| 79 | TGFB1 |  |  | 4944 | -0.861 | -0.3167 | Yes |
| 80 | PPP1R15A |  |  | 4966 | -0.882 | -0.3081 | Yes |
| 81 | SPHK1 |  |  | 4997 | -0.928 | -0.3006 | Yes |
| 82 | PDGFA |  |  | 4998 | -0.929 | -0.2873 | Yes |
| 83 | ADA |  |  | 5005 | -0.945 | -0.2750 | Yes |
| 84 | S100A10 |  |  | 5011 | -0.954 | -0.2623 | Yes |
| 85 | PROCR |  |  | 5097 | -1.139 | -0.2623 | Yes |
| 86 | PERP |  |  | 5111 | -1.186 | -0.2479 | Yes |
| 87 | CDKN2B |  |  | 5118 | -1.200 | -0.2319 | Yes |
| 88 | NUPR1 |  |  | 5123 | -1.217 | -0.2152 | Yes |
| 89 | TRIB3 |  |  | 5148 | -1.313 | -0.2011 | Yes |
| 90 | PHLDA3 |  |  | 5150 | -1.320 | -0.1824 | Yes |
| 91 | JAG2 |  |  | 5151 | -1.321 | -0.1635 | Yes |
| 92 | DDIT3 |  |  | 5180 | -1.461 | -0.1480 | Yes |
| 93 | SOCS1 |  |  | 5184 | -1.475 | -0.1274 | Yes |
| 94 | TM4SF1 |  |  | 5235 | -1.812 | -0.1111 | Yes |
| 95 | S100A4 |  |  | 5238 | -1.817 | -0.0855 | Yes |
| 96 | SLC7A11 |  |  | 5260 | -2.181 | -0.0584 | Yes |
| 97 | KRT17 |  |  | 5295 | -4.538 | 0.0000 | Yes |
Table: GSEA details [plain text format]

  

Fig 2: HALLMARK\_P53\_PATHWAY: Random ES distribution      
 Gene set null distribution of ES for **HALLMARK\_P53\_PATHWAY**
